# Supporting Holistic Wellbeing for Performing Artists During the COVID-19 Pandemic and Recovery: Study Protocol

**DOI:** 10.3389/fpsyg.2021.577882

**Published:** 2021-02-04

**Authors:** Melanie Stuckey, Véronique Richard, Adam Decker, Patrice Aubertin, Dean Kriellaars

**Affiliations:** ^1^Centre de recherche, d’innovation et de transfert en arts du cirque, École nationale de cirque, Montréal, QC, Canada; ^2^College of Rehabilitation Sciences, Rady Faculty of Health Sciences, University of Manitoba, Winnipeg, MB, Canada

**Keywords:** circus arts, human performance, psychological distress, resilience, physical literacy

## Abstract

The COVID-19 pandemic resulted in the abrupt closure of circus schools, venues, and companies, introducing a myriad of novel stressors. Performers and students must now attempt to maintain their technical, physical, artistic, creative, and cognitive abilities without in-person support from their coaches and must manage the isolation from their training and performing spaces. For circus artists, the transposition of the work space to a home environment is not possible, which creates novel stressors that could lead to the exacerbation and escalation of mental health issues. The purpose of this study is to develop, implement and evaluate a holistic interventional program based on the socio-ecological model of resilience and operationalized through physical literacy. This will be a prospective longitudinal study with a retrospective comparison to data from a similar student cohort pre-pandemic. Interventions were designed using a population-specific, participant-based developmental model within a knowledge translation framework. The interventional program includes group webinars, small group information sessions, and one-on-one Zoom meetings, in addition to the distribution of electronic educational materials. The interventions will holistically provide psychological, physical, social, technical, artistic, and creative supports. Resources will be deployed throughout the closure period and through recovery, as transitions to return to training after prolonged hiatus will magnify known psychological and physical difficulties. Repeated, longitudinal assessment of students will be utilized to track changes over time at key transitions in the pandemic and school year and will be compared to a pre-pandemic school year. The framework for this program will be translatable to other performing arts and high-performance contexts. The program has implications for the mental health and overall wellbeing of artists and for cultural and economic recovery of the industry.

## Introduction

In March 2020, performing arts training facilities, schools and professional companies in Canada were mandated to close in response to the COVID-19 outbreak. Thousands of performing artists were laid off and left uncertain about their future employment. Without proper instruction and facilities for the maintenance and development of their craft in artistic, psychological and physical terms, they risk being under-prepared to re-join the workforce when their industry re-opens. Failure to make allowances for realistic return could result in artists and technical staff seeking alternative employment pathways. While other sectors of the entertainment industry, including professional sports, are working to return to modified seasons, the performing arts industry remains largely shut down, unlikely to reopen in a conventional manner in the near future.

Mental health issues are expected to increase in the general population with the experience of the pandemic (Torales et al., 2020). For instance, increased uncertainty about the future caused by the COVID-19 outbreak has been shown to result in cognitive dissonance, negative emotions, and lower life satisfaction leading to feelings of mental discomfort (Li et al., 2020). It is important to examine the biopsychosocial impacts of the pandemic (Castelnuovo et al., 2020), which could be amplified in performing artists who face additional challenges due to their unique work and training context. Performing artists strongly rely on their bodies to express their art which becomes intimately linked, or embodied, with cognition and emotion, and vice versa (Rokotnitz, 2018). Not only is cognition embodied in performing artists, it can also be considered as embedded and extended, especially in circus artists (Bessone, 2017; Malinin, 2019) who frequently play, interact, and connect deeply with the environment (e.g., props, apparatus, and audience). When performing, some even consider their apparatus as an extended part of their cognitive system (Sevdalis and Wöllner, 2016). As such, these physical artists could be uniquely vulnerable to psychological challenges associated with the COVID-19 outbreak through the forced separation of material, social, and mental spaces, in addition to general psychological distress related to the pandemic and unemployment.

Additionally, prior to the pandemic, when compared to age- and sex-matched peers, a similar percentage of circus students were classified as having severe psychological distress (9 versus 10–13.6% normative), yet a substantially higher percentage were classified in the moderate distress category (42 versus 24–31% normative; Decker, 2020). Given the high proportion of circus students in the moderate category, the added stressors of the pandemic have the potential to shift them into the severe category, thus increasing their risk of mental distress. Furthermore, it was shown that circus students were less well adapted in their mental, social, and physical health than professionals circus artists (Donohue et al., 2018). Therefore, interventions aimed at maintaining circus skills while also maintaining mental health are important for this population.

### Psychological and Holistic Health of Circus Artists

Little is known about how the psychological experiences of circus artists compared to other performance domains such as sport. Yet, findings of a qualitative investigation revealed that mental skills such as confidence, concentration, energy management, and emotional management are crucial to support artist development (Ross and Shapiro, 2017). Since circus arts exemplifies a truly embodied cognition context, a holistic approach is required to simultaneously address cognitive, affective and performance demands (Rokotnitz, 2018). There is currently no research examining holistic health interventions in circus students and limited evidence in performance artists in general; however, leaders in the field argue that integrating interventions supporting coping, resilience, and creativity as part of the circus school curriculum is key to optimize artists’ wellbeing (Ménard and Hallé, 2014; Filho et al., 2016; Burtt and Lavers, 2017).

### Resilience

Because the pandemic situation presents multiple adversities for the performing arts community, a holistic health intervention using a resilience approach has the potential for positive impact in this population. Resilience is a multidimensional construct influenced by the intertwined relationship between the body and the mind (Jefferies et al., 2019). “In the context of exposure to significant adversity, resilience is both the capacity of individuals to navigate their way to the psychological, social, cultural, and physical resources that sustain their wellbeing, and their capacity individually and collectively to negotiate for these resources to be provided and experienced in culturally meaningful ways” (Ungar, 2008, p. 225). It involves nurturing internal resources such as self-efficacy and self-compassion (Ledesma, 2014; Masten, 2015). According to the socio-ecological model of resilience (Ungar et al., 2013), environments that provide resources to develop or maintain optimal psychological, social, and physical wellbeing facilitate the capacity of individuals to withstand, overcome, and adapt to adversity. Specifically, resilience-promoting interventions should consider the principles of *equifinality* (i.e., different interventions may produce conditions for individuals’ potential to be optimized), *differential impact* (i.e., interventions exert a different impact across individuals, time, and context) and *contextual and cultural moderation* (i.e., protective interventions are culturally and contextually grounded) (Ungar et al., 2013). According to these principles: (1) an intervention with multiple modalities is important as resilience could be influenced by various pathways, including biological, social, and environmental (Cicchetti and Blender, 2006); (2) environmental factors, including family, and community should be taken into account as they will influence the impact of the intervention on individuals (Sanders et al., 2017); and (3) the intervention needs to be appropriate to the individuals’ culture and context (Sanders et al., 2017). Importantly, addressing both internal and external resources ensures a community-based approach so the individual is not left to manage on their own (Jefferies, 2020). Interventions based on the socio-ecological model of resilience have shown positive impacts on both optimal growth and development in school and home settings (Twum-Antwi et al., 2020). This will be the first project using a resilience approach based on this model in a performing arts context. The resilience model needs to be operationalized in a manner that works in the circus context.

### Physical Literacy

**FIGURE 1:**
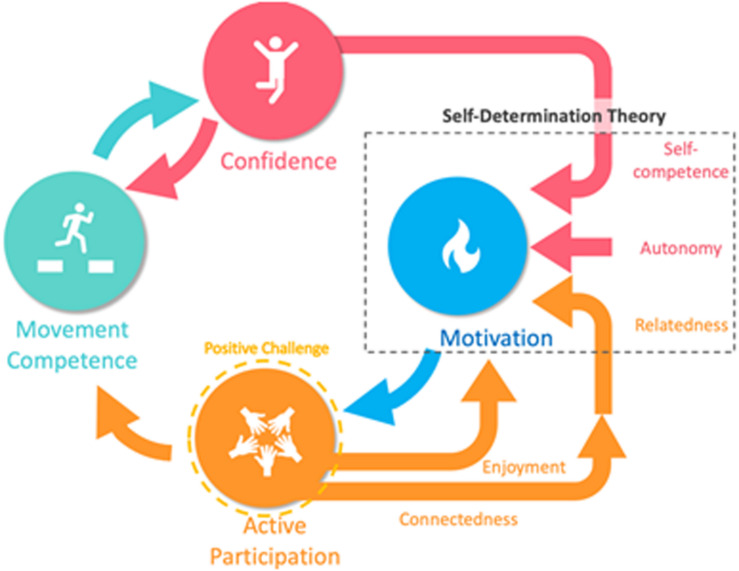
Physical literacy cycle adapted from Cairney et al., 2019 and Jefferies et al., 2019 with self-determination theory integrated.

Physical literacy offers a unique holistic approach and process (Jefferies et al., 2019) which includes crucial psychological components that are essential for maintaining and restoring competencies and capacities (Cairney et al., 2019). It also provides a putative pathway to overall wellbeing in the performance arts contexts (Cairney et al., 2019). The physical literacy process has been identified where movement competence, confidence, motivation and social participation are linked in a positive feedback cycle (Cairney et al., 2019; Jefferies et al., 2019; Figure 1). Self-determination theory can be integrated to bolster the process for self-motivation in a social context (Deci and Ryan, 2012). Additionally, this core cycle does, at least partially, address the embodied nature of performance arts (Whitehead, 2013). Importantly, strong linkages between resilience and physical literacy have been demonstrated (Jefferies et al., 2019), and recent work has demonstrated that the construction of positive challenges in the training context of circus arts pupils may be the critical element for improving resilience (Jefferies et al., 2019; Jefferies, 2020). Furthermore, circus arts have shown simultaneous positive enhancement of physical, social and psychological attributes in youth (Kriellaars et al., 2019). Enhancing the understanding of physical literacy by all the actors [artists, coaches, health care professionals, artistic staff, safety staff (riggers), etc.] involved in the circus training context may be one key element of a holistic approach to foster protective environments for artists. Further, this common resilience and physical literacy framework would serve to form a collective approach to the development and care of artists, rather than the traditionally siloed models, and may facilitate a higher level of authentic exposure and trust between artist and all staff when under pandemic duress.

### The Current Study

The impact of the COVID-19 pandemic on the performing arts industry provides a unique opportunity to examine the effects of an intervention grounded in resilience and physical literacy on the holistic health of circus students. This study may provide a foundation for translating this intervention framework to other performance and movement contexts where it can holistically address physical, psychological, social, and creative needs and emphasize resilience and overall wellbeing.

## Methods and Analysis

### Design

A prospective longitudinal design with retrospective comparison to temporally matched data from a pre-pandemic school year, since previous research showed differences between circus students and the general population, as well as the existence of temporal variation in psychological characteristics within the school training year (Decker, 2020).

### Setting and Participants

The study will be conducted at an elite-level circus training school in Montreal, Canada. The school provides high school and three-year college level programs to prepare students for a professional career in circus arts. A more detailed description of the program of study can be found in Decker et al. (2019). Since the intervention will be implemented within the school curriculum, the entire cohort of college students will be included in the intervention and will be offered the opportunity to participate in the evaluation (nominally over 110 students for the college program, age range from 16 to 27, Male:Female ratio 1.5:1).

### Intervention

**FIGURE 2:**
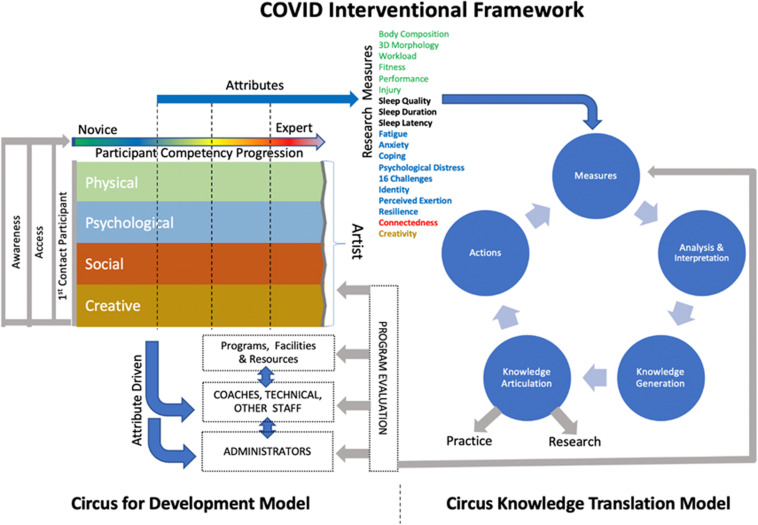
Framework to support the holistic wellbeing of performing artists.

The intervention will be deployed for one full calendar year, aligning with the end of the upcoming school year (April 2020–April 2021). In accordance with the multisystemic model of resilience and physical literacy principles, our previously developed Circus for Development Model (CfD – see Figure 2 left) will be used to guide the development of the intervention. Informed by empirical work and applied intervention done within the circus context (e.g., Ménard and Hallé, 2014; Burtt and Lavers, 2017), CfD presents a continuum of competencies to be developed through and for circus artists’ optimal growth from novice to expert. Namely, it integrates four key attributes that have been shown to contribute to artists’ performance and wellbeing, including *physical attributes* (Decker et al., 2019; Kriellaars et al., 2019; Barker et al., 2020), *psychological attributes* (Shrier and Hallé, 2011; Filho et al., 2016; Ross and Shapiro, 2017; Donohue et al., 2018; van Rens and Filho, 2019), *interpersonal and social attributes* (Filho et al., 2017; Filho and Rettig, 2018), and *creative attributes* (Leroux and Batson, 2016). These four attributes will inform the learning domains of the intervention delivered to students to ensure that their holistic development and wellness are addressed.

The CfD is connected to our internal knowledge translation framework, the Actionable Dashboard framework, which adapts Graham et al. (2006) Knowledge to Action framework to the performing arts context (Figure 2 – right). This combination creates a framework foundation for our COVID-19 intervention that necessarily includes stakeholders in continued communication with researchers to ensure the environment continues to support needs as the intervention is adapted to react to the progression of the pandemic. The program will evolve with the pandemic, be culturally grounded in circus reality, and continuously adapted to specific student needs. It will also provide specific support to key stakeholders responsible for the students’ development. Importantly, students will have access to resources including coaching (technical and artistic), safety (rigging and cleaning), healthcare (athletic therapists, medical doctor, social worker, and mental performance consultant), and educational staff, and will be supported to navigate to the resources in a timely manner.

The intervention will be delivered in phases aligning with the school year and pandemic restrictions. From April to June 2020, reactive online classes and programs were put in place to rapidly respond to the sudden temporary closure of the school and cessation of in-person training and courses. From July to August 2020 online support for students to maintain their wellbeing during the summer break will be provided. From September 2020 to April 2021 online support will be provided to students to supplement their in-facility training time, which will be significantly reduced to abide by jurisdictional limitations imposed due to the pandemic. In accordance with the contextual limitations, the program will use various delivery methods, such as (1) formalized regular communication to provide relevant information, (2) weekly interactive webinars on pre-determined specific topics, (3) weekly “open office hours” offered by specific staff, (4) online delivery of physical preparation programs, (5) one-on-one meetings for individualized support as required, and (6) identification and communication of community-based resources.

The content and topics addressed by the various facets of the program will be designed around the CfD model and will be tailored to address the needs identified by the repeated assessments. Both internal and external resources will be addressed. It is expected that the topics will include:

1) Psychological
  • Emotional regulation, uncertainty management, motivation, resiliency, and dealing with catastrophic thoughts, and substance use.
2) Physical
  • Physical maintenance, sleep, body composition, nutrition, injury prevention, artistic, and technical training.
3) Social
  • Relationship management, meaningful connections while maintaining physical distance.
4) Creativity
  • Internet-based methods of creative expression, creativity fueling, character development, acting, and entrepreneurship.

### Procedures

In a previous series of studies, a battery of health and wellbeing assessments were administered to a similar cohort of circus students at strategic timepoints within the school year: September (commencement of semester one and upon return from summer break), December (conclusion of semester one and immediately prior to technical and academic exams), January (commencement of semester two and upon return from winter break), and April (conclusion of semester two and immediately prior to summative technical and academic exams). For the COVID interventional project, students will be assessed at key milestones during the COVID pandemic, as well as time points consistent with prior measurement to allow for temporally matched comparisons to the pre-COVID status (Table 1). With the ever-changing restrictions due to the pandemic, it is challenging to predict the exact milestones that may occur during the upcoming school year and the full impact it will have on students at all levels. It is likely that all students will have a modified training year with significantly fewer 1-on-1 training hours, and the assessment schedule allows both comparison with previous years, and to assess how well students are adjusting to the changed environment.

**TABLE 1:** Rationale for post-COVID data collection points.

Time	Potential COVID-related challenges
*May 2020* Post-COVID outbreak, during school closure	• School closure • No access to training facilities
*July 2020* Post-COVID outbreak, end of school year	• Completion of spring semester (optional for some) • First session of online training; • Potential for some minimal re-opening of facilities.
*Sept 2020* Beginning of fall semester	• Potential for return to training • Extended deconditioning • Travel restrictions for international students.
*Dec 2020* End of fall semester	• Potential plan to travel home with travel restrictions • Technical assessments following first ‘COVID-modified’ semester • Potential effects of second closure with second wave of outbreak
*Jan 2021* Return from winter break	• Potential for travel issues with mandatory quarantine for international students • Possible deconditioning from 3-week winter break
*April 2021* Summative assessment for college students	• Completion of a full school year with COVID-related modifications.

### Measures

All measures will be distributed to participants via a single online questionnaire according to the schedule in Table 1. Measures were selected to align with the four domains of the CfD model (psychological, physical, social, and creative).

#### Circus Daily Challenges Questionnaire

Information regarding the daily challenges (hassles) of the students will be attained via the Circus Daily Challenges Questionnaire (CDCQ), adapted from the validated College Student-Athletes’ Life Stress Scale (CSALSS; Lu et al., 2012). The questionnaire scores each of sixteen daily challenges relevant to a developing circus artist context based on the level (intensity) of the challenge and the self-perceived difficulty to manage the challenge. The level score ranges from 0 (none) to 3 (high) and the management score ranges from 0 (no difficulty) to 2 (high difficulty).

#### Perceived Coping

The students’ evaluation of their physical and mental capacity to manage stress will be measured using a scale ranging from 1 (very poor ability) to 7 (very good ability) combined with an assessment of their perceived access to coping resources inside and outside of the school (0 = not really to 4 = very good). The four scores were summed to derive a total perceived coping score (0 – 18). The questions related to perceived coping were included as a section within the CDCQ.

#### State Anxiety

State anxiety will be assessed using a single-item (0 = no anxiety to 4 = high anxiety), based on the work of Davey et al. (2007).

#### Habits and Behaviors

A five-point scale (improved a lot, improved, not changed, slightly worse, and substantially worse) was used to assess self-reported changes in eating, technical training, physical preparation, artistic development, fitness, sleep, physical activity level, mental health, alcohol, and marijuana use in the current circumstance (measurement period) relative to the pre-COVID state.

#### Non-specific Psychological Distress

The six item Kessler Psychological Distress Scale (K6; Kessler et al., 2002) will be used to screen for moderate to severe non-specific psychological distress. K6 scores between 8 and 12 indicate moderate psychological distress, while scores equal to or greater than 13 indicate severe psychological distress.

#### Sleep and Fatigue

Sleep and fatigue metrics will be assessed using a modified version of the validated Consensus Sleep Diary (Carney et al., 2012). Sleep duration is derived from the recorded times for falling asleep and waking. Sleep quality, sleep latency, wakefulness (feeling refreshed upon waking), and fatigue will be assessed using ten-point numerical rating scales, whereby a score of one indicates a desirable score and 10 indicates an undesirable score. Students were also asked to record their daily napping behavior via a simple yes/no question each day.

#### Creativity

Two creativity assessment tools were developed to assess artists “creative state and challenges related to the pandemic situation. According to the five A’s framework ‘creativity is concerned with the action of an actor or group of actors, in its constant interaction with multiple audiences and the affordances of the material world, leading to the generation of new and useful artifacts’ (Glǎveanu, 2013, p. 76).” Building on the 5A model of creativity, both tools investigate *actors* (i.e., motivation, mindset, perception, and identity), *actions* (i.e., imagination, ideation, and exploration), *affordances* (i.e., constraints, available material, and use of the body), *audiences* (i.e., support and communication), and *artifacts* (creative outcomes). The first tool uses a five-point agreement scale from 1 (strongly disagree) to 5 (strongly agree) while the second tool, inspired by the Creative Activity Checklist (Runco and Jaeger, 2011), uses a frequency scale from 1 (never) to 5 (always).

### Data Collection and Analysis

All data will be collected electronically through the surveys and exported to Microsoft Excel, then imported to SPSS and Jamovi for statistical analysis. Between-group analysis will be used to examine differences between sexes and pre- and post-COVID (Mann-Whitney test), differences between disciplines (Kruskal Wallis), and differences between years in training program (Kruskal Wallis). Within-group analysis (Friedman test with Durbin-Conover pairwise comparison) will be used to assess variation in measures over time. Spearman correlation will be used to examine the relationships between key variables at specific times.

### Data Interpretation and Knowledge Articulation

All data will be analyzed upon receipt and presented to the school’s wellbeing committee. Following the actionable dashboard, differences between the pre- and post-COVID outbreak data will be interpreted carefully to allow for an accurate identification of consequences specifically caused by the pandemic. The assumption of consequences of such an unprecedented situation, without direct assessment of the students’ states, could misguide interventions and lower impacts. The proposed approach will, thus, generate contextualized knowledge to guide the school’s time and energy allocation to the most pressing matters to provide appropriate support as a means to optimize students’ wellbeing.

Based on the socio-ecological model of resilience, it is imperative to use data to identify resources that could be tailored to the individual (from assessments) and provide means to navigate to and negotiate for the resources that have been created (Marttila et al., 2012). One often neglected, yet important aspect for resource allocation in crisis settings is the issue of trust. Positive functioning in compromised settings requires the development of trust (Marttila et al., 2012); specific to the school’s context, trust between staff and students. The key findings will, thus, shape ongoing knowledge dissemination and presentations among these two groups to instill a community that values transparent communication, which is essential to build a trusting rapport. Furthermore, knowledge will be articulated to promote clear understanding of the reasons behind each intervention to enhance engagement.

The school has established strong partnerships over time with other performing arts schools and professional organizations. These partnerships will create a knowledge conduit, providing a multi-directional process for sharing and tailoring knowledge to the context. As knowledge is created from this applied research project, a collaborative approach will be established with partners to adapt it to local contexts, to identify barriers, to tailor the interventions to align with their artists’ needs, and, where possible, share resources and platforms to augment the collective ability. Such an approach will result in an augmented learning experience for performing arts stakeholders, allowing for more evidence-based and culturally grounded intervention programs.

The dissemination of the results will enrich the body of literature that is emerging since the COVID outbreak by highlighting the specific impacts of this unprecedented situation on the performing arts community. The comparison with longitudinal baseline data collected from a previous student cohort is a strength of the current program. It will enable the analysis to go beyond a mere description of the students’ holistic states by pinpointing specific issues that are derived from the pandemic situation. Findings will also give valuable insight and provide guidelines to partners and the performing arts community at large for establishing meaningful programs to sustain and improve artists holistic wellbeing facing massive changes in the industry. The program has the potential to significantly improve the mental and physical wellbeing of students and provides a leading-edge approach to handling pandemic circumstances. The resources invested to support the artists through this crisis will facilitate a safe, efficient transition back to training and performing, enhance holistic wellbeing, and will also facilitate the recovery of the performing arts industry, which has important implications for the economy and culture.

### Ethics and Regulatory Approval

Baseline data collection was approved by the relevant academic research ethics boards. Ethics approval for the use of the new data generated by program evaluation will be sought.

### Limitations

Analysis of the survey data may necessitate revisions and/or additions to the intervention frameworks, as these models and their use in pandemic context are not validated, but have ecological validity. Additional psychological constructs may need to be considered to align with new iterations of the framework. Furthermore, the implementation of interventions will be secondary to providing training and academic instruction related to the professional degree. Finally, there may be very different circumstances due to travel restriction for international students.

## Discussion

This applied research study will examine the effects of a holistic, resilience-promoting program based on the CfD model founded in physical literacy on the wellbeing of circus students over the course of a school year affected by COVID-19 restrictions compared to a typical school year. The goal of the intervention is to help the community thrive through the remainder of and following the pandemic. This study will provide valuable information about the mechanisms used by students to respond and rebound from adversity and will increase collective knowledge about successful interventions to enhance students’ wellbeing in a physically restricted context. Hence, the results of the current investigation could guide future intervention on how equifinality, differential impact, and contextual and cultural moderation can be addressed within a circus community to shape an environment promoting holistic wellbeing. Furthermore, combining this socioecological approach to resilience with the CfD framework to design interventions supporting artists in a time of intense adversity raises promising research and applied opportunities. Findings will provide empirical ground for the CfD framework while better defining interventions that are effective to sustain physical, psychological, social and creative competencies through adversity as exemplified next. Importantly, while the CfD model was developed specifically in circus arts, its foundation in physical literacy makes it applicable to many contexts where people engage in learning and development through movement. Our comprehensive model could have implications for human development and performance optimization in all performing arts, sport and athletics, military, rehabilitation, and health and wellbeing in the general population.

## Ethics Statement

Ethical review and approval was not required for the study on human participants in accordance with the local legislation and institutional requirements. Written informed consent from the participants’ legal guardian/next of kin was not required to participate in this study in accordance with the national legislation and the institutional requirements.

## Author Contributions

MS and VR drafted the manuscript and which was critically reviewed with significant input from all authors. All authors contributed significantly to the design of the project, analysis plan, and authorize the submission of this manuscript and accept responsibility for the publication.

## Conflict of Interest

The authors declare that the research was conducted in the absence of any commercial or financial relationships that could be construed as a potential conflict of interest.

## References

[B1] Barker L. Burnstein B. Mercer J. (2020). Acceleration profile of an acrobatic act during training and shows using wearable technology. *Sports Biomech.* 19 201–211. 10.1080/14763141.2018.1460394 29792560

[B2] Bessone I. (2017). Social circus as an organised cultural encounter embodied knowledge, trust and creativity at play. *J. Intercult. Stud.* 38 651–664. 10.1080/07256868.2017.1379962

[B3] Burtt J. Lavers K. (2017). Re-imagining the development of circus artists for the twenty-first century. *Theat. Dance Perform. Train.* 8 143–155. 10.1080/19443927.2017.1316305

[B4] Cairney J. Dudley D. Kwan M. Bulten R. Kriellaars D. (2019). Physical literacy, physical activity and health: toward an evidence-informed conceptual model. *Sports Med.* 49 371–383. 10.1007/s40279-019-01063-3 30747375

[B5] Carney C. E. Buysse D. J. Ancoli-Israel S. Edinger J. D. Krystal A. D. Lichstein K. L. (2012). The consensus sleep diary: standardizing prospective sleep self-monitoring. *Sleep* 35 287–302. 10.5665/sleep.1642 22294820 PMC3250369

[B6] Castelnuovo G. De Giorgio A. Manzoni G. M. Treadway D. C. Mohiyeddini C. (2020). Psychological, behavioral, and interpersonal effects and clinical implications for health systems of the Coronavirus (COVID-19) Pandemic: a call for research. *Front. Psychol.* 11:2146 10.3389/fpsyg.2020.02146 PMC7541698 33071842

[B7] Cicchetti D. Blender J. A. (2006). A multiple-levels-of-analysis perspective on resilience: implications for the developing brain, neural plasticity, and preventive interventions. *Ann. N. Y. Acad. Sci.* 1094 248–258. 10.1196/annals.1376.029 17347356

[B8] Davey H. M. Barratt A. L. Butow P. N. Deeks J. J. (2007). A one-item question with a likert or visual analog scale adequately measured current anxiety. *J. Clin. Epidemiol.* 60 356–360. 10.1016/j.jclinepi.2006.07.015 17346609

[B9] Deci E. L. Ryan R. M. (2012). “Self-determination theory,” in *Handbook of Theories of Social Psychology*, eds Van Lange P. A. M. Kruglanski A. W. Higgins E. T. (New York, NY: Sage Publications Ltd), 416–436. 10.4135/9781446249215.n21

[B10] Decker A. (2020). *Longitudinal Assessment of Physical Physiological and Psychological Characteristics of Elite Circus Student-Artists.* Doctoral thesis, University of Manitoba, Winnipeg.

[B11] Decker A. Aubertin P. Kriellaars D. (2019). Sleep and fatigue of elite circus student-artists during one year of training. *Med. Probl. Perform. Artists* 34 125–131. 10.21091/mppa.2019.3021 31482170

[B12] Donohue B. Gavrilova Y. Galante M. Burnstein B. Aubertin P. Gavrilova E. (2018). Empirical development of a screening method for mental, social, and physical wellness in amateur and professional circus artists. *Psychol. Aesthet. Creat. Arts* 14 313–324. 10.1037/aca0000199

[B13] Filho E. Aubertin P. Petiot B. (2016). The making of expert performers at Cirque du Soleil and the national circus school: a performance enhancement outlook. *J. Sport Psychol. Action* 7 68–79. 10.1080/21520704.2016.1138266

[B14] Filho E. Pierini D. Robazza C. Tenenbaum G. Bertollo M. (2017). Shared mental models and intra-team psychophysiological patterns: a test of the juggling paradigm. *J. Sports Sci.* 35 112–123. 10.1080/02640414.2016.1158413 26967590

[B15] Filho E. Rettig J. (2018). Team coordination in high-risk circus acrobatics. *Interact. Stud.* 19 499–518. 10.1075/is.16035.fil 33486653

[B16] Glǎveanu V. P. (2013). Rewriting the language of creativity: the five A’s framework. *Rev. Gen. Psychol.* 17 69–81. 10.1037/a0029528

[B17] Graham I. D. Logan J. Harrison M. B. Straus S. E. Tetroe J. Caswell W. (2006). Lost in knowledge translation: time for a map? *J. Continuing Educ. Health Prof.* 26 13–24. 10.1002/chp.47 16557505

[B18] Jefferies P. (2020). Physical literacy, and resilience: the role of positive challenges. *Sci. Bonheur.* 5 11–26.

[B19] Jefferies P. Ungar M. Aubertin P. Kriellaars D. (2019). Physical literacy and resilience in children and youth. *Front. Public Health* 7:346. 10.3389/fpubh.2019.00346 31803709 PMC6877541

[B20] Kessler R. C. Andrews G. Colpe L. J. Hiripi E. Mroczek D. K. Normand S. L. (2002). Short screening scales to monitor population prevalences and trends in non-specific psychological distress. *Psychol. Med.* 32 959–976. 10.1017/s0033291702006074 12214795

[B21] Kriellaars D. J. Cairney J. Bortoleto M. A. C. Kiez T. K. M. Dudley D. Aubertin P. (2019). The impact of circus arts instruction in physical education on the physical literacy of children in grades 4 and 5. *J. Teach. Phys. Educ.* 38 162–170. 10.1123/jtpe.2018-0269

[B22] Ledesma J. (2014). Conceptual frameworks and research models on resilience in leadership. *SAGE Open* 4:2158244014545464 10.1177/2158244014545464

[B23] Leroux L. P. Batson C. R. (2016). *Cirque Global Quebec’s Expanding Circus Boundaries.* Chicago: McGill-Queen’s University Press.

[B24] Li S. Wang Y. Xue J. Zhao N. Zhu T. (2020). The impact of COVID-19 epidemic declaration on psychological consequences: a study on active weibo users. *Intern. J. Environ. Res. Public Health* 17:2032 10.3390/ijerph17062032 PMC7143846 32204411

[B25] Lu F. J.-H. Hsu Y.-W. Chan Y.-S. Cheen J.-R. Kao K.-T. (2012). Assessing college student-athletes’ life stress: initial measurement development and validation. *Measur. Phys. Educ. Exerc. Sci.* 16 254–267. 10.1080/1091367X.2012.693371

[B26] Malinin L. H. (2019). How radical is embodied creativity? Implications of 4E approaches for creativity research and teaching. *Front. Psychol.* 10:2372. 10.3389/fpsyg.2019.02372 31695653 PMC6818493

[B27] Marttila A. Johansson E. Whitehead M. Burström B. (2012). Dilemmas in providing resilience-enhancing social services to long-term social assistance clients. A qualitative study of Swedish social workers. *BMC Public Health* 12:517. 10.1186/1471-2458-12-517 22789127 PMC3462131

[B28] Masten A. S. (2015). *Ordinary Magic: Resilience in Development.* New York, NY: Guilford Publications.

[B29] Ménard J. F. Hallé M. (2014). “Circus also needs performance psychology: facts and realities of consulting at Cirque du Soleil,” in *Becoming a Sport Exercise and Performance Psychology Professional: a Global Perspective*, eds Cremades J. G. Tashman L. S. (New York, NY: Psychology Press), 127–134.

[B30] Rokotnitz N. (2018). Performance and cognition: how the performing arts contribute to the science of mind. *Interdiscipl. Literary Stud.* 20 470–485. 10.5325/intelitestud.20.4.0470

[B31] Ross A. Shapiro J. (2017). Under the big top: an exploratory analysis of psychological factors influencing circus performers. *Perform. Enhanc. Health* 5 115–121. 10.1016/j.peh.2017.03.001

[B32] Runco M. A. Jaeger G. (2011). *Products*. *Advantages of the rCAB.* Available online at: http://creativitytestingservices.com/ (accessed April 16, 2020).

[B33] Sanders J. Munford R. Boden J. (2017). Culture and context: the differential impact of culture, risks and resources on resilience among vulnerable adolescents. *Child. Youth Serv. Rev.* 79 517–526. 10.1016/j.childyouth.2017.07.007

[B34] Sevdalis V. Wöllner C. (2016). “Capturing motion for enhancing performance: an embodied cognition perspective on sports and the performing arts,” in *Performance Psychology. Perception, Action, Cognition, and Emotion*, eds Raab H. M. Lobinger B. Hoffmann S. Pizzera A. Laborde S. (Amsterdam: Elsevier), 223–234. 10.1016/b978-0-12-803377-7.00014-4

[B35] Shrier I. Hallé M. (2011). Psychological predictors of injuries in circus artists: an exploratory study. *Br. J. Sports Med.* 45 433–436. 10.1136/bjsm.2009.067751 21047839

[B36] Torales J. O’Higgins M. Castaldelli-Maia J. M. Ventriglio A. (2020). The outbreak of COVID-19 coronavirus and its impact on global mental health. *Intern. J. Soc. Psychiatry* 66 317–320. 10.1177/0020764020915212 32233719

[B37] Twum-Antwi A. Jefferies P. Ungar M. (2020). Promoting child and youth resilience by strengthening home and school environments: a literature review. *Intern. J. Sch. Educ. Psychol.* 8 78–89. 10.1080/21683603.2019.1660284

[B38] Ungar M. (2008). Resilience across cultures. *Br. J. Soc. Work* 38 218–235. 10.1093/bjsw/bcl343

[B39] Ungar M. Ghazinour M. Richter J. (2013). Annual research review: what is resilience within the social ecology of human development? *J. Child Psychol. Psychiatry* 54 348–366. 10.1111/jcpp.12025 23215898

[B40] van Rens F. E. C. A. Filho E. (2019). Not just clowning around: Investigating psychological mechanisms underlying accidents in a heterogeneous group of contemporary circus artists. *Psychol. Aesthet. Creat. Arts* 10.1037/aca0000289 [Epub ahead of print].

[B41] Whitehead M. (2013). Definition of physical literacy and clarification of related issues. *J. Sport Sci. Phys. Educ.* 65 28–33.

